# In this month’s Bulletin

**DOI:** 10.2471/BLT.17.000317

**Published:** 2017-03-01

In the editorial section, Michelle J Hindin & Amanda M Kalamar (166) introduce new data sources indicating the contraceptive needs of adolescents.

Etienne Krug & Alarcos Cieza (167) make a case for integrated rehabilitation services.

Sophie Cousins (170–171) reports on efforts in Sri Lanka to maintain unprecedented reductions in malaria transmission. Vikram Patel (172–173) explains how people with depression can be treated in settings with no mental health professionals.

## Australia

### A century’s progress

Carolyn Staines & Joan Ozanne-Smith (174–181) track a long-term reduction in deaths from drowning.

## Haiti

### Ensuring good quality primary care

Anna D Gage et al. (182–190) study an approach to diagnosing performance. 

## Brazil 

### Pregnancy outcomes after maternal Zika infection

Thomas Jaenisch et al. (191–198) estimate the risk of babies having microcephaly. 

## Norway

### An antibiotic’s journey to market

Christine Årdal et al. (220–226) weigh effective antibiotic stewardship against decreased commercial incentive. 

## Nepal 

### Moving from polio eradication

Krishna P Paudel et al. (227–232) explore the feasibility of delivering other vaccines. 

## Nigeria

### Medicines that children can swallow

Ebiowei SF Orubu & Catherine Tuleu (238–240) explain why flexible solid oral formulations need to be readily available.

## Global

### Foodborne diarrhoea

Martyn D Kirk et al. (233–234) estimate the paediatric disease burden due to contaminated food.

### More scope for innovation

Erin Sparrow et al. (235–237) review potential uses for therapeutic antibodies.

### Preventing rabies

Jocelyn A Kessels et al. (210–219) advocate for pre-exposure prophylaxis in high-risk populations.

### Critical delays in receipt of vaccines

Aparna Schweitzer et al. (199–209) analyse timing of Hepatitis B vaccination across 47 countries. 

